# Accelerating PREFUL MRI: Comparison of Registration Methods and Impact of Time Series Reduction

**DOI:** 10.1002/nbm.70283

**Published:** 2026-04-09

**Authors:** A. Voskrebenzev, G. H. Pöhler, F. Klimeš, M. M. Klein, R. A. Müller, F. Wacker, J. M. Hohlfeld, J. Vogel‐Claussen

**Affiliations:** ^1^ Institute of Diagnostic and Interventional Radiology Hannover Medical School Hannover Germany; ^2^ Biomedical Research in Endstage and Obstructive Lung Disease Hannover (BREATH) German Center for Lung Research (DZL) Hannover Germany; ^3^ Department of Diagnostic and Interventional Radiology, Charité Universitätsmedizin Berlin Corporate Member of Freie Universität Berlin and Humboldt‐Universität zu Berlin Berlin Germany; ^4^ Clinic for Radiology Universital Hospital Münster, University of Münster Münster Germany; ^5^ Department of Respiratory Medicine Hannover Medical School Hannover Germany; ^6^ Fraunhofer Institute of Toxicology and Experimental Medicine Hannover Germany

**Keywords:** functional pulmonary proton MRI, image registration, optimization, perfusion/ventilation (V/Q), PREFUL (eight max), repeatability

## Abstract

The purpose of this study is to evaluate whether accelerated registration and reduced acquisition times preserve the robustness of phase‐resolved functional lung (PREFUL) MRI, thereby improving clinical feasibility by facilitating integration into tight time slots and enabling faster turnaround of results.

PREFUL MRI at 1.5 T was retrospectively analyzed in 28 patients with chronic obstructive pulmonary disease (COPD) and 56 healthy volunteers. Image registration was performed using Advanced Normalization Tools (ANTs) and Forsberg. Time series (TS) were truncated from 55 to 45, 30, and 15 s per slice. The 55‐s variant was evaluated twice to assess potential nondeterministic effects; the first served as the reference. PREFUL parameters, including regional ventilation, quantified perfusion (QQ), flow volume loop correlation metric (FVL‐CM) and corresponding defect percentages, were compared across registration methods and TS lengths using Wilcoxon tests, Pearson correlations, intraclass correlation coefficients (ICC), and regional overlap analysis. Forsberg reduced computation time by ~20‐fold (3 vs. 58 min per slice) while maintaining trends and repeatability. Although functional values were slightly lower and defect scores higher, correlations between registrations exceeded 90% for most parameters, with regional overlap ≥ 97% in healthy volunteers and ≥ 80% in COPD patients. Shortening the TS to 30 s introduced minor deviations but preserved overlap (≥ 79%) and strong correlations (≥ 77%) compared with 55 s, except for QQ. Larger deviations occurred primarily at 15 s. Repeatability remained moderate to high (ICC ≥ 68%) for all parameters, except QQ, down to 30 s, with the FVL‐CM, perfusion defect percentage and amount of nondefect regions in COPD being most stable (ICC ≥ 72%). Forsberg provides results comparable to ANTs at a fraction of the processing time, enabling PREFUL analysis within a practical timeframe. Acquisition for each slice may be reduced to 30 s without compromising robustness, supporting more efficient protocols, although caution is warranted for QQ.

## Graphical abstract

Retrospective shortening of the PREFUL MRI acquisition showed that most parameters remained strongly correlated (≥ 77%) and regionally concordant (overlap ≥ 79%) with the 55‐s ground truth down to 30 s in healthy volunteers and COPD, except for perfusion, which was more sensitive to truncation. Comparing the ANTs registration with Forsberg markedly reduced registration time (3 vs. 58 min per slice) while maintaining high correlation (≥ 90%) and overlap (≥ 80%). However, the registration method generally introduced bias.

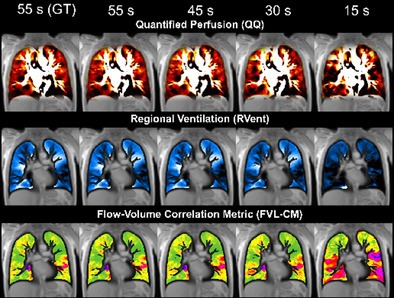

**Abbreviations**
ANTs: Advanced Normalization Tools
CF: cystic fibrosis
COPD: chronic obstructive pulmonary disease
FD: Fourier decomposition
FEV_1_
: forced expiratory volume in 1 s
FVC: forced vital capacity
FVL‐CM: flow volume loop correlation metric
GOREG: group‐oriented registration
GT: ground truth
ICC: intraclass correlation coefficients
%pred: predicted
PREFUL: phase‐resolved functional lung
QD: perfusion defect
QDP: QD percentage
QQ: quantified perfusion
ROI: region of interest
RVent: regional ventilation
TS: time series
V/Q: ventilation and perfusion
VD: ventilation defect
VDP (FVL‐CM): VD percentage based on flow volume loop correlation metric
VDP (RVent): VD percentage based on regional ventilation
VQM: V/Q match of nondefect regions

## 1 Introduction

In recent years, proton magnetic resonance imaging (MRI) has gained momentum as a promising tool for pulmonary functional imaging [1, 2]. Free‐breathing Fourier decomposition (FD) methods, in particular, enable noninvasive and contrast‐free assessment of lung ventilation and perfusion (V/Q) by exploiting temporal signal fluctuations during free breathing [3, 4, 5, 6, 7, 8, 9, 10, 11]. These fluctuations, which reflect local changes in lung volume and pulmonary blood flow, can be interpreted as surrogate ventilation and perfusion components. Although indirect, FD‐based metrics have been validated in multiple studies [12, 13, 14, 15, 16, 17, 18, 19, 20, 21, 22, 23] and shown to be clinically meaningful, offering a less costly and more patient‐friendly proton MRI alternative to more established modalities such as hyperpolarized xenon and dynamic contrast‐enhanced MRI.

Among FD approaches, phase‐resolved functional lung (PREFUL) MRI has been extensively investigated in recent years. PREFUL has demonstrated sensitivity to disease progression and treatment response in a variety of pulmonary conditions, including post–lung transplantation [23, 24, 25, 26], bronchodilator therapy in chronic obstructive pulmonary disease (COPD) [27] and asthma [28], and patients with cystic fibrosis (CF) undergoing triple combination therapy [29]. As PREFUL moves closer to clinical application and multicenter studies [21, 22, 30], two key challenges emerge: time‐efficient image registration and acquisition duration.

First, the accuracy of PREFUL critically depends on robust image registration, which aligns all frames to a uniform respiratory state. The widely used Advanced Normalization Tools (ANTs [31]) provides high‐quality diffeomorphic registration and has repeatedly performed well in benchmarking studies [32, 33] but is computationally expensive and time‐consuming. Faster alternatives such as the Forsberg registration package [34, 35] have recently been proposed and successfully tested in 3D PREFUL [36] and further comparative work in lung phantoms and COPD patients [37] has indicated registration quality comparable to ANTs. However, these findings cannot be directly transferred to 2D PREFUL, where through‐plane motion cannot be corrected and may therefore have a stronger impact on functional outcomes. Moreover, the additional spatial dimension in 3D PREFUL alters acquisition characteristics, including differences in signal‐to‐noise ratio, spatial resolution, and respiratory phase sampling and therefore may affect registration behavior such that registration performance must be empirically determined for 2D implementations. Importantly, in contrast to 3D PREFUL, 2D PREFUL relies on inflow‐related signal changes to encode perfusion, introducing additional high‐frequency temporal components that pose further challenges for image registration. Consequently, a systematic evaluation of registration performance for perfusion‐weighted metrics in 2D PREFUL is currently lacking. Addressing these limitations, our first aim is to directly compare Forsberg and ANTs within the 2D PREFUL framework and to evaluate their impact on both ventilation‐ and perfusion‐derived functional metrics.

Second, acquisition efficiency is a major concern in clinical and multicenter trial settings, where scanner time is scarce and costly. Current PREFUL protocols typically require approximately 1 min per slice, leading to total acquisition times of 3–10 min depending on lung coverage [38]. This duration may limit feasibility, particularly when PREFUL serves as a secondary endpoint. Reducing the length of the time series by half without compromising diagnostic integrity could substantially improve scalability. Related decomposition methods, such as the matrix pencil technique, have already demonstrated the potential for shortened acquisitions in a preliminary study [39], and early PREFUL results are similarly encouraging [40]. Therefore, the second aim of this study is to systematically investigate the effect of truncated time series on PREFUL‐derived metrics and their repeatability.

In summary, this study addresses two central bottlenecks for the clinical translation of PREFUL MRI: (i) validation of fast Forsberg registration versus the established ANTs framework in 2D PREFUL and (ii) systematic evaluation of time series truncation to optimize acquisition efficiency. Together, these assessments aim to examine the robustness and scalability of PREFUL for future clinical and multicenter applications.

## 2 Methods

**FIGURE 1:**
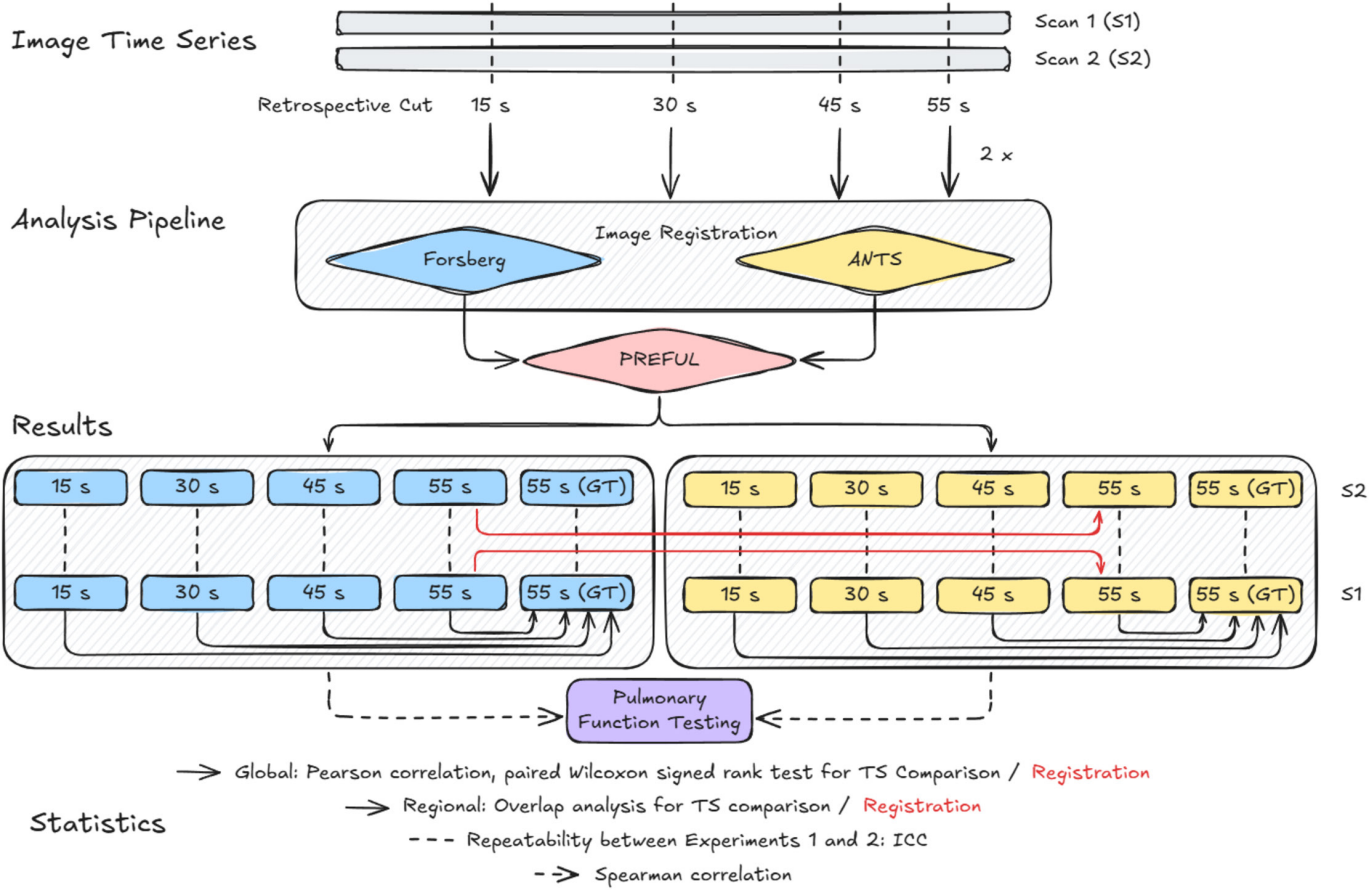
Schematic overview of the study design and analysis pipeline. Image time series from Scan 1 (S1) and the repeated Scan 2 (S2) were retrospectively truncated to 15, 30, 45, and 55 s. The 55‐s dataset was evaluated twice to account for potential nondeterministic registration effects, with the first analysis defined as the ground truth (GT). All chunks were processed with the PREFUL pipeline using two registration variants (Forsberg and ANTs). Resulting parameters were compared to the 55‐s GT using global and regional measures (black arrows) and between Ex. 1 and Ex. 2 using intraclass correlation coefficients (ICC, black dashed lines). The 55‐s datasets were additionally used to directly compare registration methods (red arrows). Finally, all results were correlated with pulmonary function test measures (black dashed arrows).

An overview of the workflow is shown in Figure 1. All statistical analyses were performed in MATLAB R2022b (MathWorks, Natick, MA, USA). The PREFUL pipeline was executed in a containerized singularity environment running MATLAB R2024a (MathWorks, Natick, MA, USA) on a high‐performance cluster. Each subject was processed as an independent Slurm batch job on a CPU‐only partition with identical resource allocation (‐‐cpus‐per‐task=12, ‐‐mem‐per‐cpu=8192, no GPU acceleration). Jobs were executed on compute nodes equipped with AMD EPYC processors. Wall clock processing times were recorded for all analyses.

Forsberg‐based registration and downstream processing were implemented in MATLAB and parallelized using parfor. ANTs registration was performed by invoking the external antsRegistration executable from within the same MATLAB parfor workflow. No method‐specific thread pinning, environment‐variable tuning, or asymmetric resource allocation was applied beyond the CPU core and memory limits imposed by the Slurm scheduler.

ChatGPT (version 5, OpenAI) was used to assist with language editing. The authors reviewed and approved all content.

### 2.1 Human Participants

Two previously published cohorts were included for retrospective analysis. The first consisted of 28 COPD patients (eight females, age range: 52–78 years) from the CLAIM study [41]. This subcohort included only patients who received placebo first, resulting in two scans 14 days apart and providing a repeatability dataset in addition to potential placebo effects. The second cohort originally comprised 57 healthy volunteers from the study by Pöhler et al. [42]; however, one dataset was unavailable in the picture archiving and communication system, leaving 56 healthy volunteers (26 males, age range: 18–66 years) for analysis. Detailed inclusion criteria and demographic characteristics are provided in the original publications. Written and informed consent was obtained from all participants during the original study procedure.

### 2.2 Image Acquisition

All participants underwent PREFUL MRI at 1.5 T (Magnetom Avanto, Siemens Healthineers, Erlangen, Germany) under free‐breathing conditions in the head‐first supine position using an eight‐channel torso phased array coil. In COPD patients, three coronal slices were acquired with the central slice positioned at the tracheal bifurcation to cover the lungs. In healthy volunteers, five coronal slices were obtained by adding one additional anterior and one posterior slice. Imaging was performed with a spoiled gradient echo sequence at a temporal resolution of 288 ms. Two hundred images per slice were acquired, resulting in a total acquisition time of 2 min and 53 s for three slices and 4 min and 48 s for five slices. Sequence parameters were TE/TR 0.82/3 ms; flip angle, 5°; matrix size, 128 × 96 (interpolated to 256 × 256); field of view, 50 × 50 cm^2^; slice thickness, 15 mm; interslice gap, 11.25 mm (distance factor 75%); and pixel bandwidth, 1500 Hz/pixel. A repeated scan was performed after 14 days in COPD patients and after a short break (~5–10 min) outside the scanner in healthy volunteers.

### 2.3 PREFUL Analysis

PREFUL analysis was performed as described in full detail by Voskrebenzev et al. [38], with the main steps summarized below.

#### 2.3.1 Simulation of Different Acquisition Times

For consistency across subjects, the full time series was truncated to 55 s (191 images) and defined as the reference length. Additional datasets of 45, 30, and 15 s were derived, corresponding to 156, 104, and 52 images, respectively. The 55‐s dataset was analyzed twice to capture potential variability from nondeterministic registration, with the first analysis designated as the ground truth (GT). Each dataset was processed once with ANTs and once with the Forsberg algorithm, resulting in 10 analyses per subject.

#### 2.3.2 Image Registration

Nonrigid registration was performed using ANTs (version 2.6.1.post1‐g4760786) and the Forsberg algorithm (commit 36c23d5). ANTs employed the SyN transform with a cross‐correlation metric (radius, 24); convergence (100 × 100 × 80 × 40, 1e‐6, 10); shrink factors, f 8 × 4 × 2 × 1; and smoothing, s 3 × 2 × 1 × 0 vox. Forsberg was configured with a polynomial expansion model and linear signal model, fluid and elastic regularization of 3, start scale of 3, six iterations per scale, multimodel set to false, symmetric set to false, and composite accumulation enabled.

Lung segmentation was performed on unregistered images using a trained nnU‐Net [43]. Respiratory phases were estimated by low‐pass filtering (cutoff, 0.7 Hz) of lung area changes, and images were sorted into 10 respiratory groups. For ANTs, group‐oriented registration (GOREG) [44] was applied: groups were intraregistered to an intermediate state, followed by intergroup registration toward Group 5 (reference). For Forsberg, initial tests showed improved performance when registering directly to the averaged image of Group 5. Parallel processing was employed to reduce computation time.

#### 2.3.3 Filtering and Preprocessing

Registered images were denoised using image‐guided filtering with the temporally averaged image as a reference. Ventilation and perfusion signals were separated with a 0.7‐Hz cutoff (low‐pass for ventilation; high‐pass for perfusion). The first 20 images were excluded from analysis to ensure magnetization steady state, except when calculating the quantitative perfusion (QQ) parameter.

#### 2.3.4 Segmentation

The temporally averaged registered images were segmented to define the lung region of interest (ROI) using a trained nnU‐Net. Large central vessels were excluded with a dedicated nnU‐Net, yielding the parenchymal ROI used for ventilation and perfusion quantification.

#### 2.3.5 Perfusion Analysis

Perfusion‐phase sorting was based on a vessel‐rich ROI identified by an iterative algorithm on perfusion‐weighted maps (standard deviation images) [20]. Seed ROIs were expanded based on fit quality, and the optimal region was selected. The vessel ROI signal was fit with piecewise sinusoidal regression, and images were sorted into 15 cardiac phases using Nadaraya–Watson kernel regression. Perfusion was defined as the signal amplitude during the parenchymal phase as determined by histogram analysis in the lung parenchyma ROI. Quantitative perfusion (QQ) was derived from transient‐state images using monoexponential fitting [45], normalized to parenchymal blood and exchange fractions, and expressed in mL·min^−1^·100 mL^−1^.

#### 2.3.6 Ventilation Analysis

Respiratory phases were estimated using the diaphragm signal after exclusion of outliers below the fifth and above the 97th percentiles. Time points were mapped to respiratory phases using a cosine‐based model, and kernel regression was subsequently applied to interpolate 60 evenly distributed phases. Regional ventilation (RVent) was calculated from signal differences between end expiration, midrespiration, and end inspiration following the Klimeš et al. method [46].

The flow volume loop correlation metric (FVL‐CM) provides an MRI analogue to spirometric flow volume analysis. RVent was first differentiated over time to obtain a surrogate of airflow. A reference loop was then defined by averaging the RVent flows within the largest connected parenchymal region with RVent values in the 80th–90th percentile. Flow volume loops of all parenchymal voxels were cross‐correlated with this reference at zero lag, and the normalized correlation coefficient was used as the FVL‐CM. Thus, lower FVL‐CM values indicate impaired or delayed ventilation relative to the reference region.

#### 2.3.7 Parameter Maps and Defect Classification

Thresholds were applied to RVent, QQ, and FVL‐CM maps according to previously established empirical rules [47]. Voxels were classified as defective if RVent was < 0.4 × 90th percentile, QQ < 0.15 × 90th percentile, or FVL‐CM < 90%. From these classifications, ventilation defect (VD), perfusion defect (QD), and V/Q match of nondefect (including both VD variants and QD) regions (VQM) were generated. Percentages of VD (RVent), VD (FVL‐CM), QD, and VQM, further referenced as VDP (RVent), VDP (FVL‐CM), QDP, and VQM, were derived based on all available slice volumes.

### 2.4 Statistical Analysis

Demographic data were summarized as median, interquartile range, and full range values. Normality was assessed using the Lilliefors test. Group differences between COPD patients and healthy volunteers were evaluated with the Mann–Whitney U test for nonnormally distributed parameters, the chi‐square test for sex, and Student's *t*‐test (equal or unequal variance depending on the result of an *F*‐test) for normally distributed parameters.

For MRI comparisons, Forsberg and ANTs registration were first evaluated on the full 55‐s datasets from both repeated scans. Global PREFUL parameters RVent, QQ, and FVL‐CM and derived defect measures were compared using paired Wilcoxon signed‐rank tests, Pearson correlation, and regional overlap after affine alignment. To assess the effect of shortened acquisitions, truncated datasets (45, 30, and 15 s) were compared with the 55‐s GT using the same tests. Repeatability of both registration methods was analyzed by calculating intraclass correlation coefficients (ICC, Type A‐1) between corresponding pairs of repeated scans.

Finally, PREFUL parameters from both registration frameworks were correlated with pulmonary function test results: forced expiratory volume in 1 s (FEV_1_), forced vital capacity (FVC), and the FEV_1_/FVC ratio, each expressed as percent predicted (%pred). Predicted values were derived from Global Lung Function Initiative reference equations using sex, age, and height [48]. Spearman's rank correlation was applied for these analyses. A schematic overview of all comparisons is provided in Figure 1. For all statistical tests described in this section, the alpha level was set to 0.05.

## 3 Results

### 3.1 Demographics and COPD vs. Volunteers Differences

Demographic information is summarized in Table 1. As expected, COPD patients were older, had a higher body mass index, and exhibited markedly reduced pulmonary function test values compared with healthy volunteers (FEV_1_ %pred: 52% vs. 92%, *p* < 0.0001). PREFUL‐derived parameters from the first scan showed analogous group differences for ANTs and Forsberg: COPD patients had lower RVent, FVL‐CM, QQ, VQM, and higher defect percentages than healthy volunteers (all *p* < 0.0001).

**TABLE 1:** Demographic characteristics of COPD patients and healthy volunteers, including pulmonary function test (PFT) results (forced expiratory volume in 1 s [FEV_1_], forced vital capacity [FVC], and corresponding predicted values) and phase‐resolved functional lung (PREFUL) MRI parameters (regional ventilation [RVent],flow volume loop correlation metric [FVL‐CM],ventilation defect percentages [VDP RVent and VDP FVL‐CM],quantitative perfusion [QQ],perfusion defect percentage [QDP],and ventilation–perfusion match of nondefect regions [VQM]) obtained with ANTs and Forsberg registration using the 55‐s GT time series. COPD patients showed the expected reduction in PFT values together with higher ventilation and perfusion defect percentages. Values are reported as median (interquartile range) (full range).

Parameter	COPD	Healthy	*p*	PREFUL	COPD		Healthy	*p*
Participants (*n*)	28	56	—	ANTs 55 s (GT)	RVent (%)	11 (08–13) [04–27]	19 (14–23) [09–45]	***†
Age (years)	65 (60–70) [52–78]	29 (23–37) [18–66]	***†		FLV—CM (%)	73 (61–80) [25–96]	98 (97–98) [93–99]	***†
Sex	20 males, 8 females	26 males, 30 females	*‡		VDP (RVent) (%)	34 (27–45) [17–69]	03 (02–06) [00–09]	***†
FEV_1_ (L)	1.44 (1.11–1.74) [0.73–2.30]	3.55 (2.91–4.14) [1.98–5.44]	*** *T*=		VDP (FVL—CM) (%)	48 (43–64) [07–91]	03 (02–06) [00–15]	***†
FEV_1_ predicted (%)	52 (40–61) [23–71]	92 (84–100) [75–119]	*** *T*≠		QQ (mL/min/100 mL)	33 (27–43) [18–99]	80 (69–99) [22–160]	***†
FVC (L)	3.17 (2.65–4.02) [2.23–5.25]	4.25 (3.56–5.64) [2.67–7.85]	***†		QDP (%)	47 (41–58) [30–71]	13 (10–17) [03–32]	***†
FVC predicted (%)	89 (81–98) [67–128]	100 (88–110) [66–145]	**†		VQM (%)	26 (12–31) [03–53]	81 (78–87) [60–94]	***†
FEV_1_/FVC (%)	43 (35–53) [23–66]	77 (74–84) [53–101]	*** *T*≠	Forsberg 55 s (GT)	RVent (%)	10 (08–12) [04–23]	17 (13–21) [08–41]	***†
Body mass index (kg/m^2^)	27 (24–30) [20–39]	23 (21–25) [17–32]	*** *T*=		FLV‐CM (%)	69 (59–78) [29–92]	97 (95–98) [92–99]	***†
GOLD 1	0	—	—		VDP (RVent) (%)	39 (30–48) [20–71]	06 (04–08) [03–13]	***†
GOLD 2	22	—	—		VDP (FVL‐CM) (%)	52 (48–67) [15–93]	05 (03–08) [00–16]	***†
GOLD 3	5	—	—		QQ (mL/min/100 mL)	30 (25–39) [15–89]	81 (72–103) [22–187]	***†
GOLD 4	1	—	—		QDP (%)	51 (41–59) [28–73]	15 (12–19) [05–32]	***†
					VQM (%)	21 (11–29) [02–53]	78 (72–83) [60–90]	***†

*Note:* Normality was assessed with the Lilliefors test; group comparisons were performed with †Mann–Whitney U test, ‡chi‐square test, *T* = Student's *t*‐test with equal variance, or *T* ≠ Student's *t*‐test with unequal variance (as determined by *F*‐test). Significance levels:

* *p* < 0.05;

** *p* < 0.01;

*** *p* < 0.001; n.s., not significant.

### 3.2 Forsberg vs. ANTs Registration

Processing times differed substantially between the two registration frameworks. Forsberg completed registration in a median of 3 min per slice for the full 55‐s dataset, compared with 58 min for ANTs. Remaining PREFUL processing steps required 5–6 min in both cases, including processing of all slices.

**FIGURE 2:**
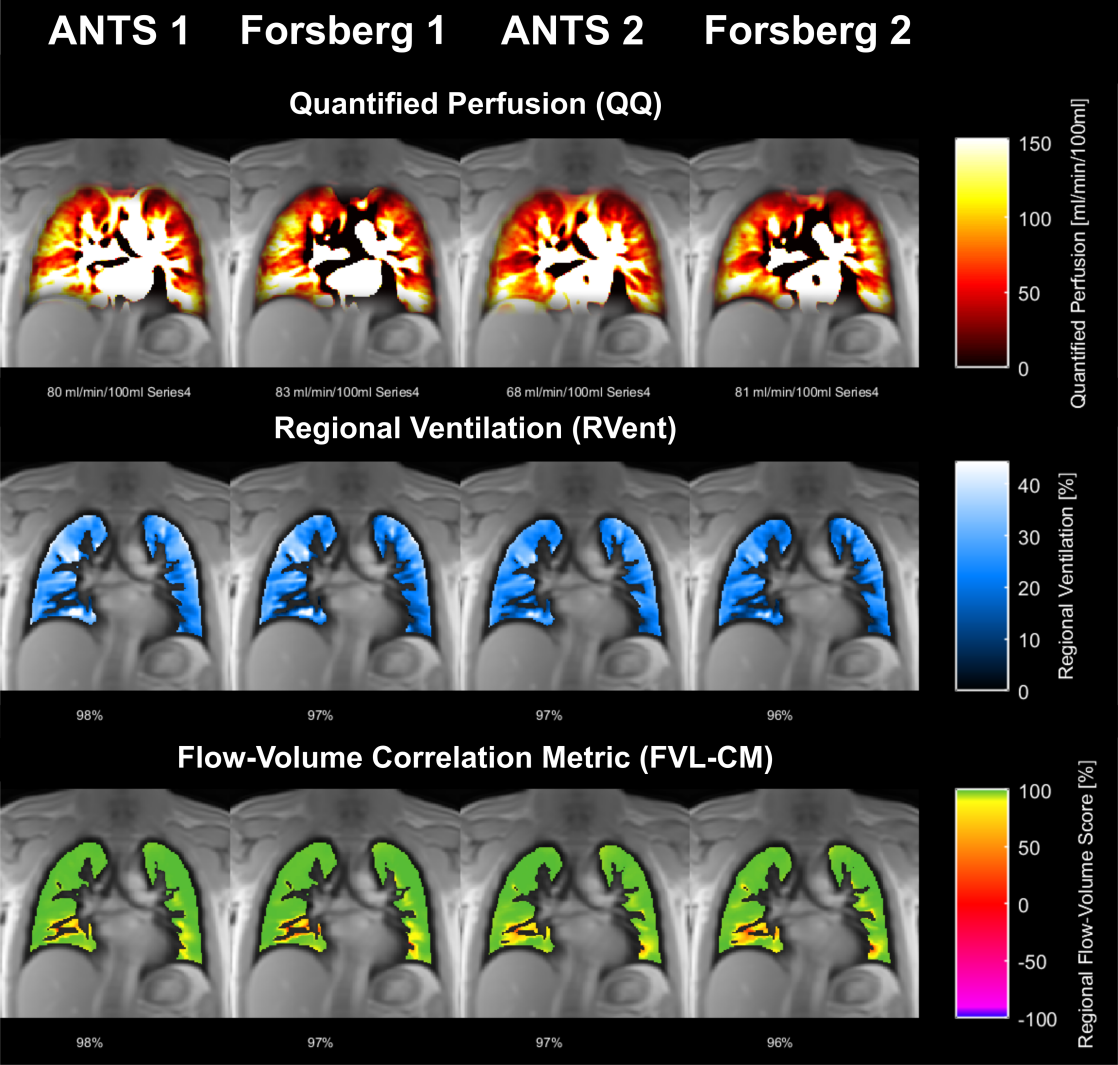
Exemplary PREFUL maps from a healthy volunteer (male, 34 years). The first two columns show results from Scan 1 using ANTs and Forsberg registration; the third and fourth columns show the corresponding results from Scan 2. A high correspondence is visible between registrations and scans, with only a minor quantitative decrease in quantified perfusion observed for ANTs in Scan 2.

Representative maps from both methods are shown in Figure 2, demonstrating good visual correspondence across scans and registrations. Quantitatively, Forsberg showed a significant but small bias towards lower absolute parameter values and correspondingly higher defect percentages, resulting in marginally reduced VQM compared with ANTs (Table 2). Nevertheless, results from both registrations were highly correlated, with correlation coefficients > 90% for most parameters. The strongest agreement was observed for RVent (100%), and the lowest for VDP derived from FVL‐CM (85%).

**TABLE 2:** Influence of ANTs and Forsberg registration on PREFUL parameters. Values are presented as median (interquartile range), with Pearson correlation coefficients and Wilcoxon signed‐rank test results reported separately for healthy volunteers and COPD patients using the first and repeated scans. Although most parameters showed significant differences between registrations, correlations remained strong, and defect maps (VDP and QDP) demonstrated high overlap.

Scan 1	Volunteers					COPD patients				
	ANTs	Forsberg	Correlation (%)	*p* (Wilcoxon)	Overlap (%)	ANTs	Forsberg	Correlation (%)	*p* (Wilcoxon)	Overlap (%)
RVent (%)	19 (14–23)	17 (13–21)	**100**	***	—	11 (8–13)	10 (8–12)	**100**	***	—
VDP (RVent) (%)	3 (2–6)	6 (4–8)	**93**	***	98 (96–99)	34 (27–45)	39 (30–48)	**99**	***	80 (76–83)
FVL‐CM (%)	98 (97–98)	97 (95–98)	**95**	***	—	73 (61–80)	69 (59–78)	**98**	***	—
VDP (FVL‐CM) (%)	3 (2–6)	5 (3–8)	**89**	***	98 (96–98)	48 (43–64)	52 (48–67)	**98**	***	87 (78–89)
QQ (mL/min/100 mL)	80 (69–99)	81 (72–103)	**91**	n.s.	—	33 (27–43)	30 (25–39)	**88**	**	—
QDP (%)	13 (10–17)	15 (12–19)	**94**	***	98 (98–99)	47 (41–58)	51 (41–59)	**98**	*	92 (87–95)
VQM (%)	81 (78–87)	78 (72–83)	**96**	***	—	26 (12–31)	21 (11–29)	**99**	***	—

Scan 2	Volunteers					COPD Patients				
	ANTs	Forsberg	Correlation (%)	*p* (Wilcoxon)	Overlap (%)	ANTs	Forsberg	Correlation (%)	*p* (Wilcoxon)	Overlap (%)
RVent (%)	18 (14–27)	16 (13–24)	**100**	***	—	12 (10–15)	11 (9–14)	**100**	***	—
VDP (RVent) (%)	4 (2–6)	7 (5–9)	**94**	***	97 (95–98)	32 (23–45)	34 (26–47)	**99**	***	82 (75–84)
FVL‐CM (%)	98 (96–98)	97 (95–98)	**94**	***	—	75 (66–86)	69 (62–82)	**98**	***	—
VDP (FVL‐CM) (%)	3 (2–6)	5 (3–8)	**85**	***	97 (96–98)	51 (29–59)	55 (36–63)	**98**	***	85 (79–89)
QQ (mL/min/100 mL)	80 (65–98)	88 (68–109)	**90**	*	—	35 (28–39)	29 (24–37)	**94**	**	—
QDP (%)	13 (9–17)	16 (11–19)	**94**	***	99 (98–99)	50 (39–60)	52 (41–61)	**99**	*	92 (87–94)
VQM (%)	82 (77–86)	77 (73–82)	**95**	***	—	22 (15–37)	20 (13–33)	**98**	***	—

*Note:* Significant Pearson correlations are highlighted in bold. Significance levels:

* *p* < 0.05;

** *p* < 0.01;

*** *p* < 0.001; n.s., not significant.

Regional overlap analyses confirmed these findings. Overlap between Forsberg and ANTs exceeded 97% in healthy volunteers and 80% in COPD patients, with the highest overlap for QDP (92%) and the lowest for VDP based on RVent (80%). Results were nearly identical across the two repeated scans.

### 3.3 Time Series Length Dependence

**FIGURE 3:**
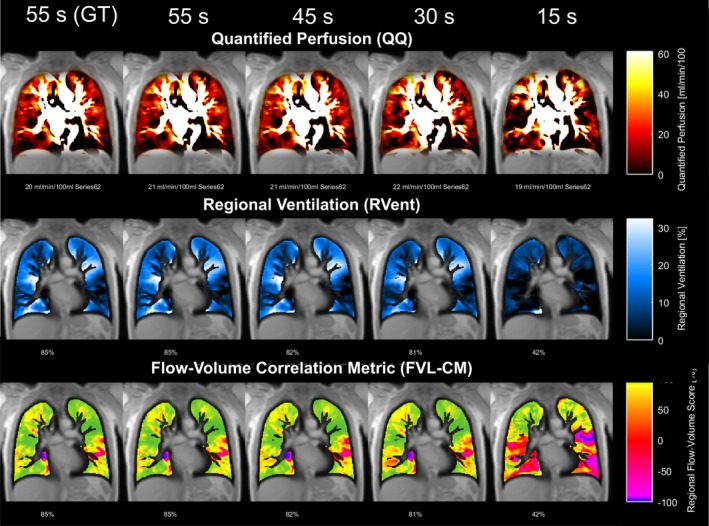
Exemplary PREFUL maps from a COPD patient (male, 70 years, GOLD 2) using ANTs registration. Each column represents results from different time series lengths. High visual and quantitative correspondence is maintained down to 30 s, with clear deviations becoming apparent at 15 s.

**FIGURE 4:**
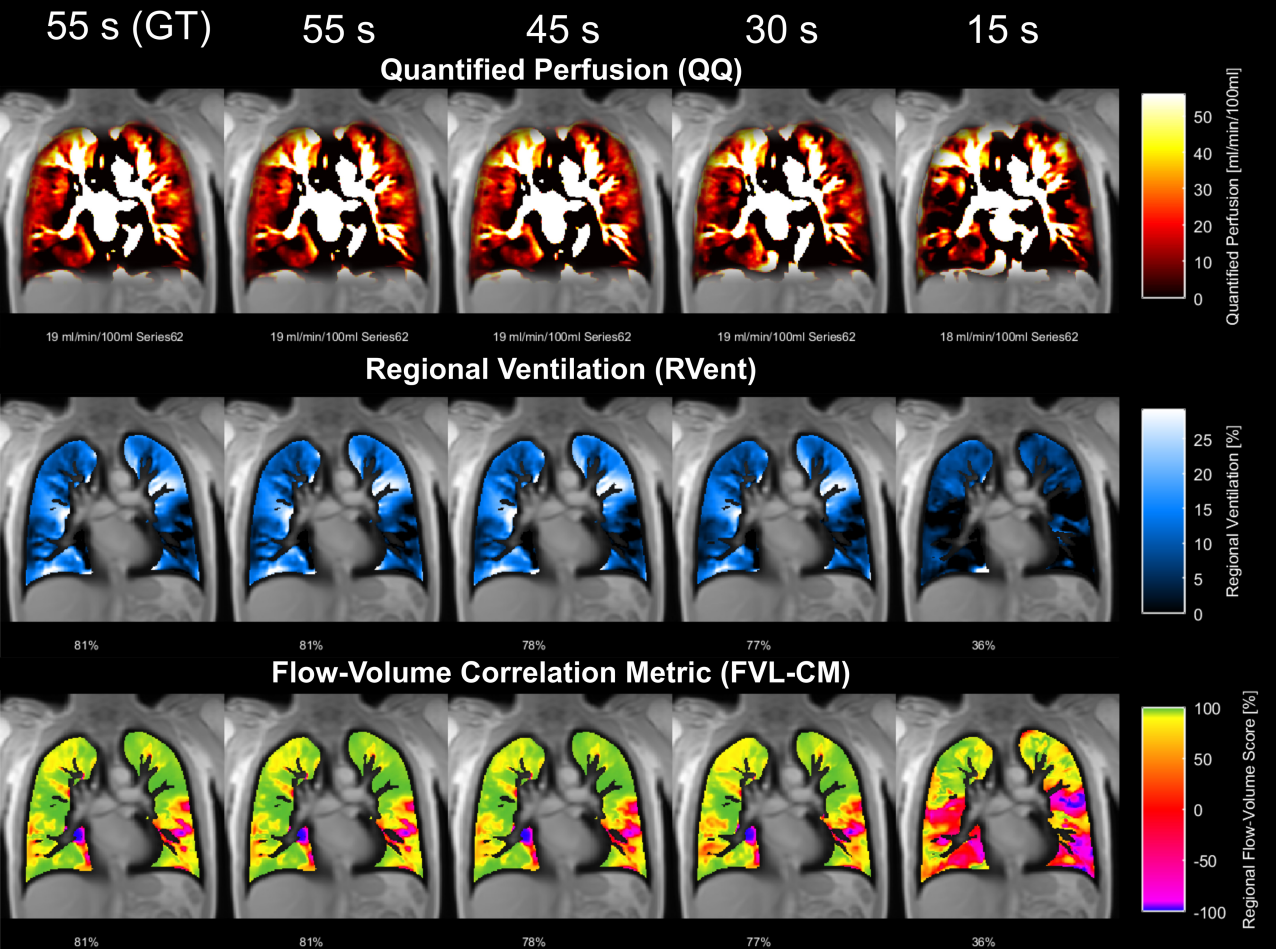
Exemplary PREFUL maps from a COPD patient (male, 70 years, GOLD 2) using Forsberg registration. Each column represents results from different time series lengths. As in Figure 3, deviations from the ground truth become noticeable at 30 s and pronounced at 15 s.

Figures 3 and 4 illustrate a representative COPD case analyzed with ANTs and Forsberg registration, respectively. In both cases, parameter correspondence remained high when the time series was shortened to 30 s, with deviations becoming evident at 15 s.

This trend was confirmed by group statistics (Tables 3, 4, 5). Mean parameter values showed significant differences from the 55‐s GT primarily at 15 s in both groups. In COPD, FVL‐CM and VDP (FVL‐CM) and RVent were significantly altered at 30 s. Forsberg produced identical results across the two repeated 55‐s analyses, with 100% correlation and matching values and ranges for all parameters. In contrast, ANTs showed small but nonsignificant variations. Nevertheless, both registration methods performed similarly when the time series was truncated. Correlations with the GT were strong for all parameters (≥ 77%) when time series length exceeded 15 s except for QQ, showing the weakest consistency (−9%, *p* = 0.5 for ANTs, and 61%, *p* < 0.001 for Forsberg).

**TABLE 3:** PREFUL results for different time series lengths with ANTs registration. Shown are median values (interquartile range), Wilcoxon signed‐rank test *p* values and Pearson correlation relative to the 55‐s GT, and intraclass correlation coefficients (ICC) with confidence intervals for repeatability across Scans 1 and 2. Results are presented separately for healthy volunteers (upper half) and COPD patients (lower half).

		Median values (interquartile range)					Wilcoxon signed‐rank *p*				Pearson correlation (%)				Repeatability ICC (%) (confidence intervals)				
		55 s (GT)	55 s	45 s	30 s	15 s	55 s	45 s	30 s	15 s	55 s	45 s	30 s	15 s	55 s (GT)	55 s	45 s	30 s	15 s
Volunteers	RVent (%)	19 (14–23)	19 (14–23)	19 (14–23)	19 (14–24)	19 (14–24)	n.s.	n.s.	n.s.	n.s.	**100**	**100**	**98**	**95**	**75 (61–85)**	**75 (61–85)**	**74 (59–84)**	**76 (62–85)**	**72 (56–82)**
	VDP (RVent) (%)	3 (2–6)	3 (2–6)	3 (2–6)	3 (2–5)	4 (2–6)	n.s.	**	**	***	**100**	**99**	**95**	**89**	**80 (68–88)**	**81 (69–88)**	**82 (71–89)**	**76 (62–85)**	**83 (73–90)**
	FVL‐CM (%)	98 (97–98)	98 (96–98)	98 (96–98)	97 (96–98)	97 (95–98)	n.s.	*	***	***	**98**	**97**	**91**	**89**	**81 (70–89)**	**83 (73–90)**	**85 (75–91)**	**75 (61–84)**	**71 (55–82)**
	VDP (FVL‐CM) (%)	3 (2–6)	3 (2–6)	4 (2–6)	4 (2–7)	6 (3–10)	n.s.	*	***	***	**96**	**96**	**88**	**85**	**72 (57–83)**	**73 (59–84)**	**79 (67–87)**	**76 (63–85)**	**69 (53–81)**
	QQ (mL/min/100 mL)	80 (69–99)	79 (68–99)	81 (69–101)	84 (70–106)	83 (69–111)	n.s.	n.s.	n.s.	n.s.	**100**	**93**	‐9	**42**	**69 (52–80)**	**69 (52–81)**	**65 (47–78)**	0 (−26–26)	**34 (8–55)**
	QDP (%)	13 (10–17)	13 (10–17)	13 (10–17)	15 (11–19)	18 (12–21)	n.s.	n.s.	**	***	**99**	**95**	**84**	**67**	**77 (63–86)**	**75 (61–84)**	**79 (67–87)**	**69 (53–81)**	**54 (32–70)**
	VQM (%)	81 (78–87)	81 (78–87)	80 (77–87)	79 (74–85)	74 (69–82)	n.s.	n.s.	***	***	**99**	**97**	**86**	**82**	**86 (78–92)**	**85 (76–91)**	**88 (80–93)**	**76 (63–85)**	**73 (58–83)**
COPD patients	RVent (%)	11 (8–13)	11 (8–13)	10 (8–13)	10 (8–13)	8 (7–11)	n.s.	n.s.	*	***	**100**	**99**	**96**	**70**	**60 (30–79)**	**60 (30–79)**	**61 (32–80)**	**74 (52–87)**	**54 (22–76)**
	VDP (RVent) (%)	34 (27–45)	35 (27–45)	39 (26–45)	39 (29–46)	42 (36–51)	n.s.	n.s.	n.s.	**	**100**	**98**	**94**	**86**	**85 (71–93)**	**85 (71–93)**	**82 (64–91)**	**69 (43–84)**	**79 (61–90)**
	FVL‐CM (%)	73 (61–80)	72 (61–80)	70 (63–79)	66 (59–79)	59 (47–72)	n.s.	n.s.	*	***	**100**	**99**	**96**	**86**	**90 (79–95)**	**90 (78–95)**	**89 (77–95)**	**82 (64–91)**	**73 (49–86)**
	VDP (FVL‐CM) (%)	48 (43–64)	48 (43–62)	52 (42–65)	52 (46–62)	62 (54–77)	n.s.	n.s.	*	***	**100**	**97**	**96**	**80**	**75 (54–88)**	**76 (55–88)**	**71 (47–86)**	**85 (70–93)**	**73 (50–86)**
	QQ (mL/min/100 mL)	33 (27–43)	33 (27–42)	33 (29–43)	38 (29–50)	40 (25–55)	n.s.	n.s.	n.s.	n.s.	**100**	**99**	**79**	**70**	8 (−31–44)	9 (−31–45)	8 (−31–44)	27 (−7–57)	25 (−17–60)
	QDP (%)	47 (41–58)	48 (41–58)	48 (40–57)	52 (37–60)	26 (16–47)	n.s.	n.s.	n.s.	***	**100**	**99**	**89**	36	**95 (89–98)**	**94 (88–97)**	**91 (82–96)**	**90 (80–95)**	31 (−7–61)
	VQM (%)	26 (12–31)	25 (13–30)	24 (13–31)	23 (14–33)	21 (15–31)	n.s.	n.s.	n.s.	n.s.	**100**	**98**	**95**	**83**	**88 (76–94)**	**88 (76–94)**	**85 (70–93)**	**85 (71–93)**	**69 (44–84)**

*Note:* Increasing deviations were observed with shorter time series. Significant Pearson correlations and ICC are highlighted in bold. Significance levels:

* *p* < 0.05;

** *p* < 0.01;

*** *p* < 0.001; n.s., not significant.

**TABLE 4:** PREFUL results for different time series lengths with Forsberg registration. Shown are median values (interquartile range), Wilcoxon signed‐rank test *p* values and Pearson correlations relative to the 55 s (GT), and ICCs (confidence intervals) for repeatability across Scans 1 and 2. Results are presented separately for healthy volunteers (upper half) and COPD patients (lower half).

		Median values (interquartile range)					Wilcoxon signed‐rank *p* (%)				Pearson correlation (%)				Repeatability ICC (%) (confidence intervals)				
		55 s (GT)	55 s	45 s	30 s	15 s	55 s	45 s	30 s	15 s	55 s	45 s	30 s	15 s	55 s (GT)	55 s	45 s	30 s	15 s
Volunteers	RVent (%)	17 (13–21)	17 (13–21)	17 (13–21)	17 (13–21)	17 (12–21)	n.s.	n.s.	n.s.	n.s.	**100**	**100**	**98**	**95**	**76 (63–85)**	**76 (63–85)**	**75 (61–85)**	**77 (64–86)**	**73 (57–83)**
	VDP (RVent) (%)	6 (4–8)	6 (4–8)	7 (5–9)	7 (5–8)	8 (5–10)	n.s.	**	**	***	**100**	**99**	**96**	**88**	**80 (68–87)**	**80 (68–87)**	**85 (75–91)**	**80 (68–88)**	**83 (73–90)**
	FVL‐CM (%)	97 (95–98)	97 (95–98)	97 (95–98)	97 (95–98)	95 (94–97)	n.s.	n.s.	***	***	**100**	**98**	**93**	**89**	**78 (66–87)**	**78 (66–87)**	**83 (73–90)**	**73 (58–83)**	**71 (56–82)**
	VDP (FVL‐CM) (%)	5 (3–8)	5 (3–8)	5 (3–7)	6 (3–9)	9 (5–13)	n.s.	n.s.	***	***	**100**	**96**	**86**	**80**	**77 (64–86)**	**77 (64–86)**	**78 (65–87)**	**73 (58–83)**	**64 (46–77)**
	QQ (mL/min/100 mL)	81 (72–103)	81 (72–103)	82 (70–106)	84 (67–106)	89 (64–114)	n.s.	n.s.	n.s.	n.s.	**100**	**93**	**61**	**53**	**50 (28–68)**	**50 (28–68)**	**38 (13–58)**	19 (−7–43)	12 (−15–37)
	QDP (%)	15 (12–19)	15 (12–19)	16 (12–20)	16 (14–21)	20 (15–24)	n.s.	*	**	***	**100**	**95**	**90**	**62**	**81 (69–88)**	**81 (69–88)**	**78 (66–87)**	**79 (67–87)**	**43 (19–63)**
	VQM (%)	78 (72–83)	78 (72–83)	77 (72–83)	76 (70–81)	70 (64–78)	n.s.	n.s.	***	***	**100**	**96**	**92**	**83**	**87 (79–92)**	**87 (79–92)**	**86 (77–91)**	**84 (74–90)**	**71 (55–82)**
COPD patients	RVent (%)	10 (8–12)	10 (8–12)	9 (8–12)	9 (7–12)	7 (6–10)	n.s.	n.s.	**	***	**100**	**99**	**96**	**73**	**58 (28–78)**	**58 (28–78)**	**60 (31–79)**	**76 (54–88)**	**53 (21–75)**
	VDP (RVent) (%)	39 (30–48)	39 (30–48)	41 (28–48)	42 (32–48)	46 (42–55)	n.s.	n.s.	*	***	**100**	**98**	**95**	**88**	**86 (71–93)**	**86 (71–93)**	**84 (67–92)**	**74 (50–87)**	**77 (56–89)**
	FVL‐CM (%)	69 (59–78)	69 (59–78)	66 (56–78)	62 (55–75)	56 (38–68)	n.s.	n.s.	**	***	**100**	**97**	**97**	**84**	**90 (79–95)**	**90 (79–95)**	**90 (79–95)**	**72 (49–86)**	**74 (52–87)**
	VDP (FVL‐CM) (%)	52 (48–67)	52 (48–67)	51 (46–68)	56 (50–67)	65 (57–81)	n.s.	n.s.	**	***	**100**	**97**	**96**	**79**	**73 (50–86)**	**73 (50–86)**	**82 (65–91)**	**82 (62–92)**	**67 (41–83)**
	QQ (mL/min/100 mL)	30 (25–39)	30 (25–39)	30 (27–41)	36 (29–45)	41 (26–54)	n.s.	n.s.	**	*	**100**	**94**	**89**	**70**	28 (−11–59)	28 (−11–59)	26 (−13–58)	**35 (0–63)**	30 (−12–63)
	QDP (%)	51 (41–59)	51 (41–59)	48 (41–58)	51 (41–58)	30 (17–45)	n.s.	n.s.	n.s.	***	**100**	**99**	**77**	28	**95 (90–98)**	**95 (90–98)**	**88 (76–94)**	**75 (53–88)**	24 (−16–56)
	VQM (%)	21 (11–29)	21 (11–29)	22 (11–30)	19 (13–31)	17 (11–28)	n.s.	n.s.	n.s.	n.s.	**100**	**98**	**96**	**88**	**89 (77–95)**	**89 (77–95)**	**89 (77–95)**	**88 (75–94)**	**66 (38–82)**

*Note:* Deviations increased with shorter time series. Significant Pearson correlations and ICC are highlighted in bold. Significance levels:

* *p* < 0.05;

** *p* < 0.01;**

*** *p* < 0.001; n.s., not significant.

**TABLE 5:** Regional overlap analysis of PREFUL results relative to the 55‐s (GT) measurement. Results are shown for healthy volunteers (left half) and COPD patients (right half), with Forsberg registration (upper half) and ANTs registration (lower half). Overlap values were high across time series lengths for defect measures VDP derived from RVent and FVL‐CM as well as for QDP.

		Healthy volunteers			COPD Patients		
		VDP (RVent) (%)	VDP (FVL‐CM) (%)	QDP (%)	VDP (RVent) (%)	VDP (FVL‐CM) (%)	QDP (%)
ANTs	55 s	100 (99–100)	100 (99–100)	100 (99–100)	99 (98–99)	98 (95–99)	99 (99–100)
	45 s	99 (98–99)	98 (97–99)	99 (98–100)	93 (90–94)	89 (86–92)	92 (89–96)
	30 s	98 (97–99)	97 (95–98)	98 (97–99)	84 (82–89)	81 (74–84)	86 (81–91)
	15 s	96 (93–97)	93 (90–95)	94 (92–97)	73 (68–83)	68 (59–74)	80 (70–88)
Forsberg	55 s	100 (100–100)	100 (100–100)	100 (100–100)	100 (100–100)	100 (100–100)	100 (100–100)
	45 s	99 (98–99)	98 (97–99)	99 (98–99)	93 (91–94)	88 (80–92)	93 (88–95)
	30 s	97 (96–98)	96 (94–98)	97 (96–98)	86 (82–89)	79 (74–82)	86 (79–90)
	15 s	94 (91–96)	91 (87–94)	93 (90–96)	75 (68–81)	67 (57–75)	79 (70–84)

### 3.4 Repeatability and Overlap

Repeatability across scans varied more strongly by parameter and time series length but was generally comparable for both registrations (ICC ≥ 72% except for RVent and QQ). QQ exhibited the lowest repeatability, with ICC values of 8%/27% in COPD patients at 55 s for ANTs/Forsberg. In contrast, FVL‐CM, QDP, and VQM showed strong and stable repeatability, maintaining ICC values ≥ 72% down to 30 s for ANTs and Forsberg. Interestingly, healthy volunteers showed slightly lower ICC for many parameters in comparison with COPD patients.

Overlap values between repeated scans decreased with shorter acquisitions but remained high overall. For time series ≥ 30 s, overlap exceeded 96% in volunteers and 79% in COPD patients for both registrations.

### 3.5 Correlation With Pulmonary Function Tests

Correlation analyses with pulmonary function test parameters are summarized in Table 6. Results were highly comparable between ANTs and Forsberg registration. No significant associations were found with FVC %pred. In contrast, strong correlations were observed with FEV_1_ %pred and the FEV_1_/FVC ratio %pred. These associations remained stable across time series lengths even down to 15 s, except for RVent, QQ, and QDP. VQM exhibited the most consistent behavior, correlating strongly with FEV_1_ %pred and FEV_1_/FVC %pred across all acquisition lengths (61%–85% for ANTs, 58%–84% for Forsberg).

**TABLE 6:** Correlation of PREFUL parameters with pulmonary function test (PFT) results from the first scan in COPD patients, using ANTs (upper half) and Forsberg (lower half) registration. No significant correlations were observed with FVC %pred, whereas the most significant and strong correlations (bold) were found for FEV_1_ %pred and FEV_1_/FVC %pred. As expected, defect parameters correlated negatively, while FVL‐CM and VQM correlated positively. Significant Spearman correlations are highlighted in bold.

		Correlation with FEV_1_ %pred					Correlation with FVC %pred					Correlation with FEV_1_/FVC %pred				
	Parameter	55 s (GT)	55 s	45 s	30 s	15 s	55 s (GT)	55 s	45 s	30 s	15 s	55 s (GT)	55 s	45 s	30 s	15 s
ANTs	RVent	32	32	32	**40**	**57**	−1	‐1	3	4	21	**38**	**38**	31	30	**53**
	VDP (RVent)	**−67**	**−67**	**−69**	**−65**	**−74**	−3	−2	−5	6	−7	**−74**	**−74**	**−74**	**−74**	**−79**
	FVL‐CM	**64**	**64**	**64**	**63**	**71**	0	−2	3	−1	9	**70**	**71**	**70**	**70**	**76**
	VDP (FVL‐CM)	**−61**	**−59**	**−56**	**−61**	**−71**	13	18	11	17	0	**−74**	**−71**	**−69**	**−77**	**−82**
	QQ	**38**	**38**	**40**	35	26	26	29	19	26	30	29	30	**40**	**41**	20
	QDP	**−62**	**−61**	**−62**	**−54**	−28	17	18	21	18	−6	**−77**	**−76**	**−80**	**−71**	−22
	VQM	**65**	**64**	**61**	**62**	**70**	−14	−18	−17	−21	−7	**82**	**81**	**79**	**85**	**81**
Forsberg	RVent	34	34	34	**41**	**56**	2	2	3	9	23	**40**	**40**	35	36	**50**
	VDP (RVent)	**−66**	**−66**	**−67**	**−67**	**−75**	3	3	2	6	−11	**−73**	**−73**	**−73**	**−75**	**−77**
	FVL‐CM	**64**	**64**	**66**	**65**	**76**	−6	−6	−3	1	14	**76**	**76**	**77**	**73**	**78**
	VDP (FVL‐CM)	**−57**	**−57**	**−59**	**−62**	**−69**	21	21	12	9	0	**−72**	**−72**	**−71**	**−78**	**−82**
	QQ	**39**	**39**	**48**	**44**	23	1	1	7	8	19	**38**	**38**	**49**	**42**	15
	QDP	**−57**	**−57**	**−60**	**−56**	−18	25	25	27	9	4	**−78**	**−78**	**−80**	**−66**	−17
	VQM	**58**	**58**	**57**	**61**	**63**	−28	−28	−28	−14	−12	**81**	**81**	**81**	**79**	**84**

## 4 Discussion

### 4.1 Main Findings

This study yielded two main findings. First, Forsberg registration produced results highly comparable with ANTs, as shown by strong correlations and high regional overlap, even though systematic shifts toward slightly lower functional values and higher defect scores were present. Crucially, Forsberg reduced computation times by a factor of 20, enabling PREFUL analysis within ~15 min for three slices and thereby making clinical workflows feasible.

Second, acquisition times could be shortened substantially. Although shortening altered parameter values slightly, a 30‐s time series preserved correlations, repeatability, and regional overlap for all major PREFUL parameters except for quantified perfusion, with significant deviations emerging mainly at 15 s. Thus, acquisition times can be halved without advanced acceleration techniques, supporting more efficient protocols while maintaining robustness.

### 4.2 Comparison With Previous Work

Our results align well with earlier investigations of registration comparison between ANTs and Forsberg. Klimeš et al. showed in 3D PREFUL that Forsberg achieved results equivalent to ANTs with strong correlations to pulmonary function tests [36]. Voskrebenzev et al. demonstrated comparable performance in a lung model with GT deformation while also reporting lower functional values and higher VDP for Forsberg [37]—consistent with our findings. Although these studies provide an important benchmark, their direct transfer to 2D PREFUL is not straightforward. First, in 2D implementations, through‐plane motion cannot be corrected and may therefore exert a stronger influence on functional outcomes, and the absence of an additional spatial dimension alters acquisition characteristics such as signal‐to‐noise ratio, spatial resolution, and respiratory phase sampling, all of which may affect registration behavior. Secondly, while the lung model study provided valuable controlled validation, it did not capture the full complexity of phase‐resolved 2D PREFUL post‐processing and acquisition. The present work therefore complements and extends prior evidence by evaluating registration performance in 2D PREFUL under clinically relevant acquisition and processing conditions, including both ventilation and perfusion.

Within this 2D framework, we observed better performance of a direct registration strategy for Forsberg compared with the group‐oriented approach employed in 3D. This may be explained by the lower number of groups used in 3D or greater interpolation errors from joining repeated warping steps in our 2D implementation. Notably, the group‐oriented scheme cannot account for ANTs' slower runtime: the additional registrations are equivalent to the number of groups minus one (e.g., ~10) and are therefore negligible relative to the total number of images. The observed runtime gap is more plausibly driven by algorithmic differences and their implementation—such as diffeomorphic optimization and similarity metrics.

Our findings on time series truncation are also concordant with prior work. Bauman et al. observed deviations below 24 s [39], especially for perfusion, and Munidasa et al. reported increasing ventilation defects with acquisitions shorter than 36 s in CF patients [40]. Our results support 30 s as a practical lower limit and again highlight the greater vulnerability of perfusion, likely due to its smaller amplitude and higher frequency compared with respiration.

### 4.3 Factors Influencing Robustness

Several additional observations provide insight into PREFUL stability. Forsberg consistently produced lower absolute values and higher defect scores than ANTs. Although this precludes direct interchangeability, the strong linear correlations show that both can be applied if used consistently within a study. Similarly, truncation introduces bias and must be considered when comparing across different acquisition lengths.

Shorter acquisitions led to additional ventilation and perfusion defects. We hypothesize that although partly truncation artifacts, these defects are not entirely random: Regions with already reduced function may be disproportionately affected when fewer time frames are available, as reduced functional amplitudes are more easily obscured by noise in a lower SNR setting. In this sense, truncation may act like applying a stricter threshold. The fact that repeatability did not decline monotonically with shorter series, together with high correlations to GT and PFT, supports the notion that nonrandom effects contribute to defect inflation. The sharp decline at 15 s for most metrics, however, suggests that random effects begin to dominate below 30 s.

Repeatability differed strongly between parameters. QQ showed the lowest repeatability, reflecting the complexity of its calculation (e.g., including heart rate) and its dependence on the selected normalization region, which was inconsistent between scans based on randomized visual checks. Landmark‐based approaches—for example, deep learning–based detection of the left pulmonary artery [49] as implemented by Wernz et al.—could therefore potentially improve QQ's stability. By contrast, VQM, QDP, and FVL‐CM were among the more stable metrics down to 30 s. VQM's and QDP's robustness is expected as both are threshold‐based, whereas FVL‐CM's stability likely derives from the high SNR of RVent used to define the reference region.

An apparent paradox was observed in the repeatability analysis: healthy volunteers showed partially lower ICC values than COPD patients, despite having a shorter pause between scans. This is explained by the ICC's sensitivity to variance structure. Healthy subjects form a more homogeneous group with lower between‐subject variability, which artificially lowers ICC values even when absolute repeatability is high. In contrast, COPD patients are more heterogeneous, inflating between‐subject variance and thus ICC.

Finally, we observed minor variability across repeated ANTs runs on the same dataset, attributable to random sampling and floating point precision [50]. However, this effect was negligible (correlations, ≥ 96%) and can be mitigated completely by fixing random seeds and restricting to single‐threaded computation, as suggested by the ANTs developers. Forsberg produced identical results by default. Also, its computational efficiency remains a clear advantage, though GPU‐accelerated ANTs may mitigate this gap [51].

### 4.4 Clinical Implications

PREFUL‐derived parameters showed clinically plausible associations with spirometry for both registrations. Specifically, FEV_1_ and especially the FEV_1_/FVC ratio correlated positively with FVL‐CM and the fraction of healthy voxels (VQM) while correlating negatively with defect percentages (VDP and QDP). These relationships mirror COPD pathophysiology: Preserved airway function corresponds to homogeneous V/Q patterns, whereas reduced FEV_1_ is linked to widespread functional defects. Importantly, most of these associations remained detectable even at 15‐s time series lengths, underlining the robustness of PREFUL parameters against acquisition shortening.

Our findings also demonstrate strategies for clinical translation. By markedly reducing postprocessing time, Forsberg makes PREFUL analysis more compatible with routine clinical workflows. Reducing acquisitions to 30 s doubles efficiency without sacrificing robustness, making whole lung coverage in less than 10 min achievable. This positions 2D PREFUL closer to applications where full coverage is crucial—for instance, in CF—particularly because 2D PREFUL provides both ventilation and perfusion, whereas current free‐breathing noncontrast 3D methods focus mainly on ventilation.

Furthermore, the results emphasize the need for harmonization. Both registration method and acquisition length affect the absolute value and range of PREFUL metrics, and prior studies also reported variation across sequence types and field strengths [47, 52]. Although distributed pipelines can be standardized, acquisition differences between vendors and field strengths will remain. Vendor‐independent frameworks such as pulseq may help harmonize protocols [53, 54], and when this is not feasible, transformation equations or deep learning–based harmonization approaches [55] may provide alternatives.

### 4.5 Limitations and Future Directions

This study has several limitations. Only patients with COPD were included, and all data came from a single‐center, single‐vendor setting. Results may differ in other diseases or in multicenter studies. In particular, pediatric acquisitions may be more sensitive to data truncation because of pronounced out‐of‐plane motion; in such cases, a full acquisition ensuring at least 30 s of usable data may be advisable.

Only one imaging protocol (spoiled gradient echo at 1.5 T) was tested; although previous work suggests feasibility at 3 T and with balanced steady‐state free precession sequence [47, 52], the performance under time series truncation may differ. Hyperparameter optimization of registration, filtering, and thresholding adapted to time series length was not explored and could allow for more stable performance at even shorter acquisitions.

Additionally, the 55‐s acquisition was used as the gold standard; the potential benefits of longer acquisitions remain untested. No external reference, such as hyperpolarized MRI or CT, was available to further validate whether PREFUL metrics lose spatial accuracy at shorter acquisition times. Nevertheless, previous studies have established the validity of full‐length (~1 min) PREFUL results.

It should be noted that combining MATLAB parfor execution with externally multithreaded executables may introduce nested parallelism, which can influence absolute performance measurements. However, both registration approaches were executed under identical job‐level resource constraints and within the same execution model. Consequently, runtime comparisons should be interpreted in the context of the specified computational setup, and further method‐specific optimization of parallelization strategies may yield additional runtime improvements. Finally, emerging generative AI approaches may enable the synthesis of extended time series from shorter acquisitions, offering a complementary strategy for further acceleration.

## 5 Conclusion

Forsberg registration delivers results comparable to ANTs at a fraction of the computational cost, enabling PREFUL processing within a clinically practical timeframe. Acquisitions may be shortened to 30 s without compromising robustness, preserving repeatability and overlap for key parameters. These strategies substantially improve the feasibility of PREFUL MRI and support its integration into larger clinical studies and ultimately routine practice. Nevertheless, quantitative perfusion requires particular caution, and harmonization of protocols and postprocessing is essential for consistent results.

## Author Contributions

Conceptualization: J.V.‐C. and A.V. Data curation: A.V. Formal analysis: A.V. Funding acquisition: J.V.‐C., F.W., and J.M.H. Investigation: G.H.P. and J.M.H. Methodology: A.V. and J.V.‐C. Project administration: J.V.‐C. and A.V. Resources: J.V.‐C., F.W., and J.M.H. Software: A.V., F.K., M.M.K., and R.M. Supervision: J.V.‐C. Validation: F.K., M.M.K., and R.M. Writing – original draft: A.V. Writing – review and editing: All Authors.

## Funding

This work was supported by the German Center of Lung Research (DZL).

## Conflicts of Interest

A.V., F.K., and J.V.C. are shareholders of BioVisioneers GmbH, a company that has interest in pulmonary magnetic resonance imaging methods. J.M.H. declares grants to his institution for this investigator‐initiated trial from Boehringer Ingelheim Pharma GmbH & Co. KG; grants to his institution for clinical trial conduct from Altamira, Astellas, AstraZeneca, Bayer AG, Boehringer Ingelheim Pharma, CSL Behring, Desitin Arzneimittel, EpiEndo, F. Hoffmann‐La Roche, Genentech, OM Pharma, Novartis, ReAlta Life Sciences, and Sanofi‐Aventis Deutschland; and personal fees for consultancy and board activities from Boehringer Ingelheim Pharma, Celerion, CSL Behring, Cureteq, Novartis, and Roche.

## Data Availability

The data that support the findings of this study are available on request from the corresponding author. The data are not publicly available because of privacy or ethical restrictions.
